# Mesenchymal Stem Cell-Derived Extracellular Vesicles in Liver Immunity and Therapy

**DOI:** 10.3389/fimmu.2022.833878

**Published:** 2022-03-04

**Authors:** Ruiqi Wu, Xiaoli Fan, Yi Wang, Mengyi Shen, Yanyi Zheng, Shenglan Zhao, Li Yang

**Affiliations:** Sichuan University-University of Oxford Huaxi Joint Centre for Gastrointestinal Cancer, Department of Gastroenterology and Hepatology, West China Hospital, Sichuan University, Chengdu, China

**Keywords:** extracellular vesicles, mesenchymal stem cell, immunomodulation, liver diseases, drug delivery system

## Abstract

Mesenchymal stem cells (MSCs), as the most common cell source for stem cell therapy, play an important role in the modulation of innate and adaptive immune responses and have been widely used in clinical trials to treat autoimmune and inflammatory diseases. Recent experimental and clinical studies have shown that MSC-derived extracellular vesicles (MSC-EVs) can inhibit the activation and proliferation of a variety of proinflammatory cells, such as Th1, Th17 and M1 macrophages, reducing the secretion of proinflammatory cytokines, while promoting the proliferation of anti-inflammatory cells, such as M2 macrophages and Tregs, and increasing the secretion of anti-inflammatory cytokines, thus playing a role in immune regulation and exhibiting immunomodulatory functions. Besides MSC-EVs are more convenient and less immunogenic than MSCs. There is growing interest in the role of MSC-EVs in liver diseases owing to the intrinsic liver tropism of MSC-EVs. In this review, we focus on the immunomodulatory effects of MSC-EVs and summarize the pivotal roles of MSC-EVs as a cell-free therapy in liver diseases, including NAFLD, AIH, acute liver failure, liver fibrosis and hepatic ischemia–reperfusion injury. Moreover, we provide a concise overview of the potential use and limits of MSC-EVs in clinical application.

## Introduction

Liver diseases, with their high morbidity and mortality rates, pose a significant risk to human health and, consequently, a heavy economic burden (1). Stimulation such as viral hepatitis, drugs, and alcohol abuse can trigger chronic/acute liver injury and inflammation that can lead to liver failure, cirrhosis, and even hepatocellular carcinoma (2). Currently, treatments are limited for many liver diseases, such as non-alcoholic fatty liver disease (NAFLD) and acute liver injury. In regard to cirrhosis and liver failure, orthotopic liver transplantation is the only effective treatment (3). Therefore, novel and effective treatments are urgently needed.

As nonhematopoietic pluripotent stem cells, MSCs are capable of self-renewal and differentiation. Numerous studies have shown that MSCs can effectively inhibit the activation of innate immune system cells, including macrophages (4), dendritic cells (DC) (5), natural killer cells (NK) (6), monocytes (7) and others. MSCs also inhibit the activation of adaptive immune cells, including T cell subsets (8) and B cell subsets (9). Thus, MSCs show a significant effect on immune modulation. As the central metabolic organ and important immune participant of the human body, the liver has a high density of myeloid and lymphoid immune cells, and many liver diseases are related to the destruction of liver immune homeostasis (10). Recent clinical studies have demonstrated that MSC therapy alleviates liver damage, improves liver function, and promotes liver tissue regeneration (11, 12).

Currently, it is generally believed that MSCs exhibit immunomodulatory functions mainly through the paracrine pathway, among which, extracellular vesicles (EVs) are the most appealing components. EVs are vesicles secreted by cells with phospholipid bilayer membrane, and are capable of mediating intercellular communication by transferring membrane and cytosolic proteins, lipids, and RNAs (13). Compared with MSCs, MSC-EVs are much smaller in size and easier to obtain and store. When injected intravenously *in vivo*, MSC-EVs mainly accumulate in liver (14), and liver diseases have close relationship with immune homeostasis imbalance, which fits perfectly with the function of MSC-EVs. Therefore, the scientific community has examined the application of MSC-EVs instead of MSCs in liver diseases. In this review, we summarize the effects of MSC-EVs on liver immunity and diseases, outline the limits of MSC-EVs in clinical application, and propose potential applications in the future.

## Overview of MSCs

Mesenchymal stem cells (MSCs), also mentioned as multipotent stromal cells or mesenchymal stromal cells, are multipotent cells present in almost all forms of postnatal organs and tissues (15). MSCs were initially discovered by Alexander Friedenstein approximately 50 years ago (16), and were originally named by Caplan in 1991 (17). In early clinical experiments, MSCs were mainly isolated from bone marrow, cartilage, and fat tissues (18), recently, they were also isolated from the umbilical cord, placenta, muscle, dental pulp, liver, and other tissues (19). Although MSCs from different origins share similar marker profiles and cell phenotypes, the epigenetic characteristics of MSCs from different tissues show significant differences in gene expression patterns, proteome, and function, which are only related to their source (20, 21). Without stimulation, cultured MSCs uniformly showed the morphology of plastic adherent cells (fibroblast-like cells), and expressed stromal surface markers (such as CD73, CD90, and CD105), but lacked hematopoietic cell markers [such as CD11a, CD14, CD19, CD45 and human leukocyte antigen (HLA)-DR]. Consequently, they can differentiate into adipogenic, chondrogenic, and osteogenic lineages (22).

Several preclinical studies have shown that MSCs can modulate the activation and function of various immune cells in the innate and adaptive immune systems. However, the immunomodulatory capabilities of MSCs are not constitutive and depend on the exposed inflammatory environment. In the presence of high levels of proinflammatory cytokines [such as interferon-γ (IFN-γ) and tumor necrosis factor-α (TNF-α)] or activation of Toll-like receptor 3 (TLR3) ligands, MSCs mediate immunosuppression (23). For example, MSCs inhibit the proliferation and activation of T cells (24), induce the proliferation of regulatory T (Treg) cells (6), inhibit the proliferation of stimulated B cells and their differentiation into antibody producing cells (plasma blasts) (25), and promote polarization towards an immunomodulatory M2 phenotype (26). On the other hand, during the early phase of inflammation with low levels of inflammatory cytokines or activation of TLR4 by low levels of lipopolysaccharide (LPS), MSCs show proinflammatory effects through the secretion of chemokines that recruit lymphocytes to the sites of inflammation. Conclusively, MSCs show immunosuppressive functions in the vigorous inflammatory environment (27).

Due to the functions of immune modulation and promotion of cell survival, as well as easy access and efficient proliferation *in vitro*, MSCs have been increasingly used as cell therapy in clinical trials focused on tissue repair and regeneration. However, contrary to predictions, a large number of preclinical studies have shown that MSCs are not transplanted into tissues in significant numbers after infusion, while the duration of transplantation is insufficient (28, 29). Currently, numerous evidence supports the therapeutic and immunomodulatory functions of MSCs partly but significantly dependent on paracrine effects and secretory components (30, 31). Thus, researchers turned their attention to paracrine secretions such as EVs.

## Overview of Extracellular Vesicles

Extracellular vesicles(EVs)are particles naturally released from the cell, delimited by a lipid bilayer and cannot replicate (32). They can be classified into two major categories, ectosomes, and exosomes. Ectosomes are plasma membrane-derived vesicles ranging from 50 nm to 1 mm in diameter. In contrast, exosomes originate from endosomes and are in a size range of ~40 to 160 nm in diameter (~100 nm on average) (33). According to the position statements of the International Society for Extracellular Vesicles (MISEV2014 (34) and updated MISEV2018 (32)), evidence of endosomal biogenesis, such as findings of EVs caught in the act of release by live imaging techniques, is required to classify the extracellular vesicles in the research as an “exosome”. Therefore, ISEV recommended other classifications based on physical characteristics or biochemical composition. Depending on the size of vesicles, those < 100 nm or < 200 nm are classified as “small EVs” (sEVs) and those > 200 nm are classified as “medium/large EVs” (m/l EVs), among which, sEVs are considered to be more consistent with the definition of therapeutic EVs. Therefore, we use “extracellular vesicles (EVs)” as an acronym for “exosomes” in this review. Their membranes are rich in cholesterol, sphingomyelin, and hexose ceramide, especially highly mannosylated epitopes, lectins and tetraspanin-enriched microdomains, compared with parent cells (35–37). EVs contain large amounts of proteins, such as heat-shock proteins (Hsp70 and Hsp90) (38), genetic elements, such as mRNAs and miRNAs (39), and cytokines such as TNF-α, interleukin (IL)-2, IL-6, IL-8, IL-10, etc (40). By transmitting their contents through receptor–ligand interactions, endocytosis or direct fusion to the cell membrane, EVs can mediate and regulate a variety of cellular processes in target cells (41).

### Functions of MSC-Derived Extracellular Vesicles in Immunity

**Figure 1:**
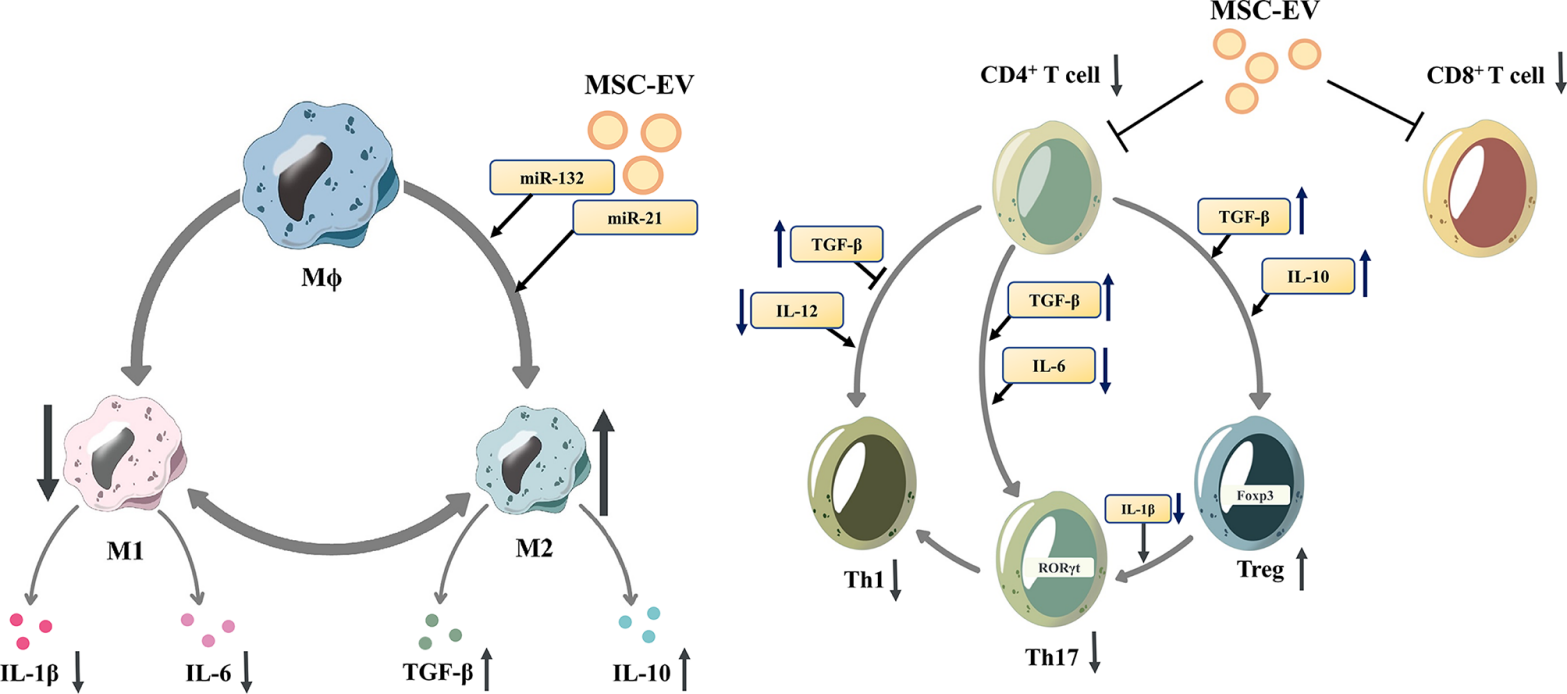
Mesenchymal stem cell-derived extracellular vesicles modulate the differentiation of macrophages and T cells. Mesenchymal stem cell-derived extracellular vesicles (MSC-EVs)reduce the proliferation of proinflammatory cells (M1 macrophages, CD8+ T cells, Th1 and Th17 cells)and downregulate the release of IL-1β and IL-6. In contrast, MSC-EVs increase the proportion of anti-inflammatory subtypes (M2 macrophages and Tregs), and upregulate the release of anti-inflammatory cytokines, such as TGF-β and IL-10. M1 macrophage, M1-polarized phenotype macrophage; M2 macrophage, M2-polarized phenotype macrophage; Th 1 cells, T helper 1 cells; Th17 cells, T helper 17 cells; Tregs, regulatory T cells; IL-1β, Interleukin 1β; IL-6, Interleukin 6; TGF-β, transforming growth factor-β; IL-10, Interleukin 10.

EVs are found in nearly all body fluids and are released by almost all types of cells in the human body, and can be isolated from cells, plasma, tissue sources, milk and even plants. Among them, cell-derived EVs are the most easily obtainable in large quantities. MSCs are one of the most easily obtained primary stem cells in the human body and secrete large amounts of EVs, which makes MSCs one of the ideal sources of EVs. In addition to CD107, CD63 and CD81, markers for EVs, MSC-EVs also express markers for MSCs, such as CD29, CD73, CD90 and CD44 (42). MSC-EVs play an important role in the regulation of the immune system. MSC-EVs carry bioactive molecules, such as mRNAs and miRNAs, cytokines, chemokines, immunomodulators, and growth factors, thereby modulating the balance between the immune response and immune tolerance (**Figure 1**).

MSC-EVs can influence innate immune cells such as macrophages, DCs and NK cells, thus modulating the innate immune response. Depending on the specific stimulus, macrophages polarize into different phenotypes, express different markers, and perform different functions. Murine macrophages can be classified into the M1 phenotype and M2 phenotype. While for human macrophages, their various phenotypes form a continuous spectrum without clear boundaries and they can switch into other phenotypes under different stimulations (43). For murine macrophages it is currently believed that TLR ligands (such as LPS) and proinflammatory cytokines (such as IFN-γ and TNF-α) induce the differentiation of M1 macrophages, while Th2 cytokines, such as IL-4 and IL-13, induce the differentiation of M2 macrophages (44). M2 macrophages produce anti-inflammatory cytokines such as transforming growth factor β (TGF-β), IL-1 receptor antagonist (IL-1RA), and IL-10 (45). MSC-EVs can promote the M2-type polarization of macrophages to regulate the immune response, which is related to the miRNAs contained, such as miR-132 (46), miR-21 (47), and miR-21-5p (48).MSC-EVs can inhibit the maturation of DCs, reduce the secretion of proinflammatory factors (49).. MSC-EVs are rich in miR-21-5p, which can reduce the migration of DCs to the CCR7 ligand CCL21, and is associated with reduced expression of CCR7 protein and decreased production of proinflammatory cytokines (50). Moreover, MSC-EVs can also inhibit the function of NK cells (51) and inhibit the proliferation of peripheral blood mononuclear cells (PBMCs) (52).

In addition, MSC-EVs control the proliferation and differentiation of T cells and B cells, modulating the adaptive immune response. T cells have multiple subsets, promoting or inhibiting the process of inflammation, coordinating multiple aspects of adaptive immunity (53). Under inflammatory stimulation, MSCs can secret EVs with activated TGF-β, which can inhibit T cell proliferation, especially CD4+ T cells through Smad and other pathways (54–57). MSC-EVs are rich in HLA-G5, which can inhibit the proliferation of CD4+ and CD8+ T cells and reduce the release of IFN-γ (58). Matrix metallopeptidase derived from MSC-EVs cuts IL-2 receptor α (CD25) on the surface of activated T cells, thereby downregulating IL-2 signaling and inhibiting T cell proliferation (59). Additionally, MSCs-EVs may promote the differentiation of naive T lymphocytes into cells with anti-inflammatory phenotypes, such as Tregs (60–62). TGF-β from MSC-EVs was also involved in the promotion of Treg proliferation (63, 64). Zhang et al. demonstrated that antigen-presenting cells (APCs) mediate the enhancement of Treg differentiation *in vitro* and *in vivo* by MSC-EVs (31, 65). In addition, MSC-EVs reduced pathogenic Th17 amplification and secretion of proinflammatory factors such as IL-17A, IL-21, IL-22, and IL-2 (66, 67). Regulatory B cells (Bregs) are subtypes of B cells that suppress immune responses, such as reducing pathogenic T helper cells (TH17 and TH1 cells), while promoting Treg cell proliferation (68). MSC-EVs increase the number of Bregs and promote IL-10 secretion (60, 69). In addition, MSC-EVs can directly inhibit the proliferation and activation of B cells (9, 70, 71).

### Liver Immunity

To clarify the immunomodulatory effects of MSC-EVs in liver diseases and explore other potential mechanisms, the immune environment of the liver should be determined. The liver is the central organ of metabolism and immune modulation. Most of the blood entering the liver comes from the portal vein rather than the hepatic artery. Blood enters the liver parenchyma mainly through the peripheral vessels of the portal vein, and then leaves the parenchyma *via* the central hepatic vein through the sinusoidal network (72). Hepatic sinuses are anatomic sites that regulate immune homeostasis in the liver. The blood flow is slow in the hepatic sinuses, which prolongs the contact between lymphocytes and antigen-presenting cells and promotes lymphocyte exosmosis. In addition to parenchymal cells (hepatocytes and cholangiocytes), there are also nonparenchymal cells [such as hepatic stellate cells (HSCs) and hepatic sinusoidal endothelial cells (LSECs)] and a large number of immune cells (such as T cells, macrophages, dendritic cells, etc.) in the liver.

The liver innate immune system is modulated by multiple cellular components; Kupffer cells(KCs), DCs, NKs, NKTs and LSECs. During liver injury or infection, KCs phagocytize the pathogenic materials and release a large number of cytokines and other products to initiate acute inflammation with the rise in acute-phase proteins (APPs). APPs include C-reactive protein (CRP), serum amyloid A (SAA), complement components and others, and they play an important role in the innate immune response with their haemostatic, microbicidal and phagocytic functions (73). KCs are resident macrophages in the liver that can bind and trap pathogens in hepatic sinuses, accounting for 80-90% of tissue macrophages (74). When stimulated and activated, KCs can regulate the phenotype of their own and other immune cells. DCs are also important in innate immune response. There are four major subsets of DCs in the liver, including myeloid CD8a (–) B220 (–), lymphoid CD8a (+) B220 (–), plasmacytoid CD8a (–) B220(+), and natural killer dendritic cells with a CD8a (–) B220 (–) NK1.1(+) phenotype (75). Both myeloid and plasmacytoid subsets can initiate innate immune responses. KCs and DCs are the first to detect pathogens and can recruit other immune cells such as monocytes and neutrophils into the liver (74). NK cells are important effectors of the innate immune system with their functions of lysing target cells and producing large quantity of cytolytic granules in the absence of prior antigen sensitization. NK cells in human blood can be divided into two subsets, CD56bright and CD56dim, and CD56dim is more in circulation. In contrast, the human liver is enriched in CD56bright NK cells that localize within hepatic sinusoids (76). During liver inflammation, NK cells are recruited early by chemokines expressed from KCs or LSECs, then secrete cytokines such as IFN-γ, promoting the progress of inflammation, or directly killing target cells such as virus-infected hepatocytes or hepatocellular carcinoma (77). Natural killer T (NKT) cells are enriched in the liver and are divided into two main subsets (type I or invariant NKT cells and type II or diverse NKT cells). Type I NKT cells are more prevalent in murine liver and Type II NKT cells are more abundant in human liver (78). NKT cells are capable of recognizing lipid antigens presented by CD1d, then release Th1 (IFN-γ, TNF-α), Th2 (IL-4, IL-5, IL-10) or Th17 (IL-17, IL-22) cytokines activating other innate immune cells and adaptive T cells (79). Type I NKT cells mainly show a proinflammatory effect in liver injury (78). In contrast, type II NKT cells were able to inhibit the proinflammatory response induced by type I NKT cells, thus preventing liver injury. LSECs are the most common nonparenchymal cells in the liver and form the vascular wall of sinusoidal cortex with open fenestrae and lack of a basement membrane. Unlike other endothelial cells, LSECs highly express scavenger receptors and mannose receptors, giving them a strong endocytosis ability. Accordingly, LSECs can directly ingest hepatitis viruses such as HBV and HCV (80, 81).

Adaptive immunity has a crucial role in controlling liver inflammation. Naive T cells are primed by APCs, such as KCs and DCs, then proliferate and differentiate into effector cells. T cells are divided into CD4 T cells, CD8 T cells and γδ-T cells according to their phenotypes and functions. CD4 T cells can be divided into proinflammatory subgroups such as Th1、Th2、Th17 and follicular helper T (Tfh) cells (82, 83), and Tregs, which usually suppress the inflammation (84). CD8 T cells include cytotoxic T cells (Tc), the main cell killer in adaptive immunity, and CD8 Treg cells, which suppress immune responses (85). The functions of γδ-T cells are phagocytosis and tumor killing, but also immune regulation (86).

Blood in the portal vein is full of nutrients and accompanying antigens from the gastrointestinal tract. Therefore, immune tolerance is required for the hepatic immune system. Hepatic APCs are core to the immune tolerance of the liver by their capacity of suppressing adaptive immunity. Under steady-state conditions, myeloid DCs, and plasmacytoid DCs produce massive anti-inflammatory cytokines such as IL-10 and TGF- β, inducing Tregs and tolerogenic T-cell responses in the liver (87). KCs also play a pivotal role in liver immune tolerance. KCs can inhibit T cell activation by secreting prostaglandins such as Prostaglandin E2 (PGE2)and 15deoxy-Δ12,14-prostaglandin J2 (15d-PGJ2) (88), while this inhibition can be reversed by pathogen-related molecules (such as TLR3) or inflammatory cytokines (89).

Moreover, liver immune regulation was also associated with damage-associated molecular patterns (DAMPs) such as high mobility group protein 1(HMGB1) and IL33 (90, 91), NOD-like receptor family, pyrin domain containing 3(NLRP3) (92), TLRs (TLR3, TLR4, TLR7, TLR9) (93) and complements (94).

In general, there is immune tolerance in the liver to prevent unwanted inflammatory responses. However, when the level or context of antigens changes, the liver can switch from immune hyporesponsiveness rapidly to an active state of generating inflammatory responses. This immune regulation involves the functional diversity of macrophages and dendritic cells, the ratio of different types of T cell subsets (such as Th17 and Treg), the balance between pro-inflammatory and anti-inflammatory cytokines, and other immune-related factors.

### Therapeutic Application of MSC-Derived EVs in Liver Diseases

**Figure 2:**
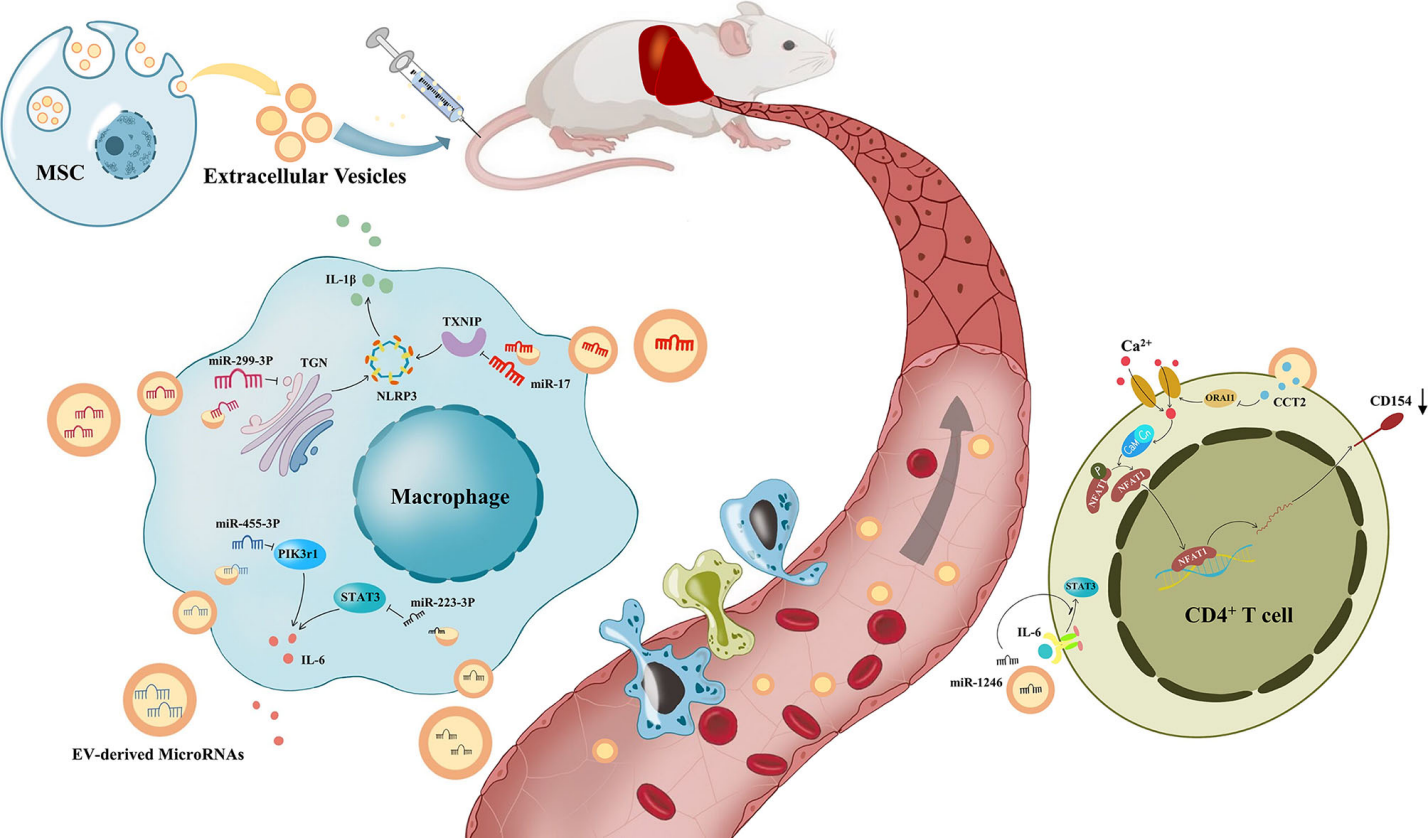
The process of immune regulation by mesenchymal stem cell-derived extracellular vesicles on the liver. Under the modulation of Mesenchymal stem cell-derived extracellular vesicles(MSV-EVs), macrophages reduce the release of IL-6 and IL-1β, and CD4+ T cells reduce the expression of CD154. Liver inflammation was improved by the immune modulation of MSC-EVs, which alleviates different kinds of liver diseases.

In this section, we have summarized the preclinical studies of MSC-EVs as a cell-free therapy in liver diseases, including NAFLD, autoimmune hepatitis (AIH), acute liver failure, liver fibrosis and hepatic ischemia–reperfusion injury, focusing on the immunomodulatory effects of MSC-EVs (**Table 1**, **Figure 2**).

**Table 1:** The role of MSC-EVs in the modulation of liver immunity in various liver diseases.

Type of disease	Immune Cell	Pathway	Effects	Reference
NASH	Macrophage	STAT3—ARG-1	polarizing M2 macrophages and producing related ARG-1 and IL-10	(95)
AIH	Macrophage	miR-223-3p—STAT3—IL-1β, IL-6	reducing Treg/Th17 ratio	(96)
Acute liver failure	Macrophage	miR-17—TXNIP/NLRP3	reducing NLRP3 inflammasome activation	(97)
Acute liver failure	Macrophage	miR-299-3p—TGN—NLRP3	reducing NLRP3 inflammasome activation	(98)
Acute liver failure	Macrophage	miR-455-3p—PIK3r1	blocking the activation of the IL-6 signaling pathway	(99)
Liver Fibrosis	Macrophage	LPS/TLR4—NF-κB	downregulating the expression of TNF-α、IL-1β and IL-6	(95)
IRI	CD4+T	CCT2—Ca2+‐calcineurin‐–NFAT1	downregulating CD154 expression	(100)
IRI	CD4+T	miR-1246—IL-6—gp130—STAT3	enhancing the shift of Th17 toward Treg cells	(101)

#### Non-Alcoholic Fatty Liver Disease

Non-alcoholic fatty liver disease (NAFLD) is a continuum of liver abnormalities ranging from nonalcoholic fatty liver disease (NAFL) to nonalcoholic steatohepatitis (NASH). At present, NAFLD is a critical issue of liver diseases, and its incidence has increased globally, reaching 24% in the latest statistics (102), posing a significant economic burden (103). NAFLD is closely related to chronic inflammation caused by stimuli both inside and outside the liver, and this inflammation is closely associated with macrophages and neutrophils (104). KCs secrete TNF, CCL2, and CCL5 to mediate monocyte infiltration, promoting steatosis and subsequent fibrosis progression (105). In addition, toxic lipids stored in KCs participate in the early stage of inflammation (106). Neutrophils seem to be observed in most liver inflammation. Focusing on NAFLD, the proinflammatory effects were based on myeloperoxidase (MPO) (107). In contrast, DCs play a protective role in NAFLD (108). NAFLD has a high incidence but limited treatments, mainly depending on body weight loss. Currently, there are many drugs targeting NAFLD in clinical trials, such as the farnesoid X receptor (FXR) agonist obeticholic acid; however, none of them have shown certain therapeutic effects (109). Thus, some researchers began to pay attention to other potential treatments.

Abrogation of liver inflammation might be achieved by MSC-EV treatment. At present, many studies have reported that mesenchymal stem cell-derived exosomes can improve liver inflammation, reduce alanine aminotransferase (ALT) and aspartate aminotransferase (AST) levels, and alleviate liver fibrosis *in vivo* (95, 110, 111). Recent studies have shown that the immunotherapeutic effect of MSC-EVs on NAFLD mainly depends on macrophages. LPS promotes the process of inflammation in NASH by activating KCs through the TLR4 signaling pathway (112). However, MSC-EVs prevented this promotion, especially the activation of M1 macrophages, and downregulated the expression of inflammatory cytokines, such as TNF-A, IL-1β and IL-6 in NASH models. In the LPS/TLR4 pathway, myeloid differential protein (MyD) 88 is activated, leading to TNF receptor associated factor 6(TRAF6) activation and phosphorylation of p65 and IκB-α, allowing NF-κB to translocate to the nucleus. MSC-EVs could suppress the phosphorylation of p65 and IκB-α, and the transcriptional activity of NF-κB, but they did not suppress the NF-κB transcriptional activity induced by overexpression of TRAF6 in HEK293 cells. These results reveal that MSC-EVs may inhibit the early steps of the LPS/TLR4 signaling pathway (110). In addition, MSC-EVs increased the number of CD11b + F4/80 + Ly6c low anti-inflammatory macrophages in the liver of mice with NASH (111). F4/80+ macrophages have relatively high phagocytosis activity and therefore actively participate in the elimination of dead white blood cells, pathogens and cell debris, promoting the regression of inflammation (113). MSC-EVs contain p-STAT3, which can bind to STAT3 target DNA and promote the transcriptional activation of the ARG-1 promoter/enhancer, thereby polarizing M2 macrophages and producing related ARG-1 and IL-10 (95).

In addition to regulating immune homeostasis, MSC-EVs can also regulate the metabolic homeostasis of NASH (95) and the apoptosis and autophagy of hepatocytes. El-Derany and colleagues demonstrated that MSC-EVs reduce apoptosis of hepatocytes, but activate mitochondrial autophagy by upregulating miRNA-96-5p expression and downregulating caspase-2 expression in HFD-induced NASH (114). He et al. showed that MSC-EVs promoted autophagy *via* the AMP-activated protein kinase (AMPK) pathway in L-O2 cells, resulting in the improvement of glucose and lipid metabolism (115).

#### Autoimmune Hepatitis

Autoimmune hepatitis (AIH) is a progressive autoimmune liver disease that is characterized by liver inflammation, circulating autoantibodies and hypergammaglobulinemia (116). Clinically, the diagnosis of AIH mainly depends on liver biopsy by observing interface hepatitis, rosettes, and lymphocyte invasion. The current first-line therapy is standard immunosuppressive treatment using corticosteroids with or without azathioprine (117). Without effective controls of inflammation, AIH may result in rapid progression to liver fibrosis, cirrhosis, and even hepatocellular carcinoma. The pathogenesis of AIH involves molecular mimicry, immune activation and loss of immune tolerance (118). CD4+T cells appear central to AIH immune mechanisms. When immune responses to external pathogens occur, recognition of autoantigens with similar structures also occurs through molecular mimicry, such as cytochrome P450 2D6 (CYP2D6) (119). Consequently, these autoantigens will be processed and presented to uncommitted naive CD4+ T helper (Th0) cells through abundant APCs in the liver, such as DCs, macrophages, and B cells. Stimulated by various cytokines, Th0 cells differentiate into Th1, Th2 and Th17 cells and then produce a large amount of proinflammatory factors. Through these processes, T cells promote the occurrence and persistence of liver inflammation. Tregs are quantitatively impaired in AIH, and this impairment is more pronounced during active disease (120). This imbalance in the number and function of Tregs and T effector cells leads to the loss of immune tolerance, which eventually results in AIH.

MSC-EVs are capable of promoting liver immune homeostasis and relieving liver inflammation by reversing the decline in Tregs in AIH. Tamura et al. reported that the necrotic area of the liver was significantly reduced and ALT elevation was inhibited in mice with Concanavalin A (ConA)-induced AIH. In addition, MSC-EVs significantly decreased the expression of the proinflammatory cytokine IL-2, while enhancing the mRNA expression of anti-inflammatory cytokines, such as TGF-β and hepatocyte growth factor (HGF), and this effect was correlated with the amount of MSC-EVs injected. Moreover, the number of Tregs was increased. They speculated that KCs produced cytokines such as TGF-β after engulfing MSC-EVs and then promoted the production of Tregs, thus inhibiting liver inflammation caused by ConA (121). Lu and colleagues also discovered that liver inflammation, ALT and AST levels were significantly decreased in mice with ConA-induced AIH after MSC-EV treatment. However, they did not observe an increase in the number of Treg cells, and they found that the ratio of Treg/Th17 cells increased instead. They reported that miR-223-3p in MSC-EVs could reduce the LPS-induced elevation of the inflammation-related genes STAT3 and p-STAT3 in macrophages, which is an important upstream activator of IL-1β and IL-6. STAT3 is known to activate RORt and promote IL-17 secretion by Th17 cells, IL-1β converts Treg cells into Th17 cells, and IL-6 can promote the differentiation of naive T cells into Th17 cells. Therefore, the proportion of Tregs/Th17 cells increased after MSC-EV treatment, which alleviated AIH (96). Moreover, miR-223 in MSC-EVs was demonstrated to attenuate NLRP3 and Caspase-1 levels in AML cells, thus reducing pyroptosis of hepatocytes (122).

#### Acute Liver Failure

Acute liver failure (ALF) is defined as a syndrome of severe injury to liver cells that leads to altered coagulation and mentation without the basis of chronic liver disease (123). Viral hepatitis, drug-induced liver injury, alcohol consumption and autoimmunity are the main causes of acute liver failure. Its clinical manifestations include hepatic encephalopathy, jaundice, coagulation dysfunction, and so on. Due to the amelioration of liver transplantation and intensive care, outcomes for patients with acute liver failure have improved, however, more effective treatments are needed despite liver transplantation.

ALF is closely related to macrophages (10). DAMPs released from dead cells can bind to pattern recognition receptors on macrophages, subsequently inducing the production of cytokines and chemokines and then activating inflammasomes, such as NLRP3, leading to the recruitment of neutrophils and mononuclear derived macrophages into the liver, which is demonstrated in the process of ALF (124). On the other hand, KCs release IL-10 and remove apoptotic materials for protection (125). Numerous studies have shown that MSC-EVs can inhibit the proliferation and activation of proinflammatory macrophages, subsequently reducing the secretion of cytokines such as IL-1β, IL-6, IL-18, and TNF-αand therefore significantly improving ALF (97–99, 126, 127). Researchers proved that the mechanism could be related to the inhibition of the NLRP3 pathway by MSC-EVs (97, 98, 126). In addition, MSC-EVs have shown their effectiveness in reducing apoptosis of hepatocytes, which is regarded as an important pathological manifestation of early-stage ALF (128–131). Some researchers believe that the reduction in apoptosis results from the activation of autophagy in hepatocytes through MSC-EVs (128). Others believe that MSC-EVs attenuate apoptosis due to the alleviation of hepatic oxidant injury (129, 130). Moreover, MSC-EVs play an important role in promoting hepatic regeneration. MSC-EVs can induce the conversion of hepatocytes into hepatic oval cells, which have the ability to proliferate in large quantities (132). Tan et al. discovered that the expression of proliferation proteins (PCNA and cyclin D1) was higher in the MSC-EV-treated group, which suggests that MSC-EVs upregulate the priming-phase genes of hepatocytes (131). Damania et al. proved that the ratio of residual liver to estimated liver weight was increased through MSC-EVs in both partial hepatectomy and ischemia–reperfusion injury-induced liver failure mouse models (133).

#### Liver Fibrosis

Most chronic liver diseases, such as viral liver disease, NAFLD, and alcoholic liver disease, can progress to fibrosis. Liver fibrosis is the replacement of damaged normal tissue by fibrous scarring resulting from the accumulation of extracellular matrix (ECM) proteins (mainly cross-linked collagen type I and type III) (134). Myofibroblasts are the main source of ECM in liver fibrosis, and hepatic stellate cells (HSCs) and portal vein fibroblasts can be transformed into myofibroblasts. HSCs can be activated in response to injury, upregulate the expression of α-smooth muscle actin(α-SMA), secrete collagen type I, migrate to the site of injury, and secrete ECM to produce fibrous scars. β-catenin and cytokines such as TGF-β can effectively stimulate the activation of HSCs and promote the transcription of collagen type I (135, 136). Macrophages play dual roles in liver fibrosis. On the one hand, they can produce cytokines and chemokines such as TGF-β to activate HSCs and enhance liver fibrosis. On the other hand, they can attenuate liver fibrosis by anti-inflammation and secretion of matrix metalloproteinases (MMPs), such as Mmp9 and Mmp12, to degrade and phagocytose existing ECM (137). It is widely believed that M1-type macrophages can develop liver fibrosis by promoting the propagation of inflammation, while M2-type macrophages attenuate fibrosis by promoting cell proliferation and reducing apoptosis of hepatocytes. However, recent studies have found that M1-type macrophages may also be involved in the process of fibrosis regression (138).

Recently, numerous preclinical studies have demonstrated the antifibrotic effect of MSC-EVs in the lung (139), liver (140), kidney (141), and heart (142). Focusing on liver fibrosis, MSC-EVs promote liver tissue repair by reducing inflammation, therefore alleviating fibrosis. MSC-EVs upregulated the expression of MMPs (143), such as MMP9 and MMP13, and anti-inflammatory factors, such as TGF-β1 and IL-10 (140, 143), downregulated the expression of pro-inflammatory factors such as TNF-α, IL-1β and IL-6, and TGF-β (110). In addition, MSC-EVs can improve CCL4-induced liver fibrosis by inhibiting epithelial-to-mesenchymal transition (144). Moreover, MSC-EVs were shown to inhibit HSC activation. Rong et al. reported that MSC-EVs can inhibit the activation of HSCs through the Wnt/β-catenin pathway *in vivo* and *in vitro* (145). Kim et al. demonstrated that miR-486-5p derived from MSC-EVs directly binds to the 3’ untranslated region (UTR) of SMO mRNA and inhibits its expression, resulting in the inactivation of HSCs (146). Wu et al. showed that miR-150-5p was poorly expressed and CXCL1 was highly expressed in liver fibrosis, while MSC-EVs could transfer miR-150-5p to HSCs, subsequently inhibiting the activation of HSCs by downregulating CXCL1 expression (147).

#### Ischemia–Reperfusion Injury

Ischemia–reperfusion injury (IRI), including ischemic organ injury and inflammation-mediated reperfusion injury, is an important cause of liver injury in surgical procedures including hepatectomy and liver transplantation. Many factors are involved in the process of IRI, such as the inflammatory response, reactive oxygen species (ROS), apoptosis, and autophagy (148). The immune response to IRI includes the activation of KCs, DCs, NK cells, and T cells, as well as the involvement of various cytokines and inflammasomes (149, 150).

Zheng et al. found that CCT2 in MSC-EVs downregulated the expression of CD154 on CD4+ T cells in the liver through the restrictive Ca2+ - calcineurin - NFAT1 pathway (100). The interaction of CD154 and CD40 stimulated innate and adaptive immune responses, and it has been proven that CD154 on CD4+ T cells plays a pivotal role in liver IRI (151). Therefore, MSC-EVs alleviate liver ischemia/reperfusion injury. miR-1246 derived from MSC-EVs could bind GSK3β in hepatocytes, significantly inhibit the expression of Wnt1, Wnt3a and β-catenin, activate the Wnt/β-catenin signaling pathway, and reduce the IRI induced production of TNF-α, IL-6, and IL-1β, thereby improving IRI by reducing inflammation (152). In addition, miR-1246 in MSC-EVs further upregulated TGF-β1 and IL-10, and downregulated IL-6 and IL-17 (101). TGF-β alone induces the differentiation of naive CD4+ T cells into Treg cells, whereas it induces differentiation into Th17 cells in collaboration with IL-6, which is controlled by the IL-6-gp130-STAT3 signaling pathway. As a result, miR-1246 improves liver IRI by regulating the balance between proinflammatory Th17 cells and anti-inflammatory Treg cells. In addition, hepatic infiltration of macrophages and neutrophils could be significantly reduced by treatment with MSC-EVs (153). Moreover, the role of MSC-EVs in apoptosis and autophagy has also been considered. In contrast, Bo et al. suggested that the reduction in apoptosis through MSC-EVs was based on the enhancement of autophagy (154).

## Therapeutic Advantages of MSC-EVs Over MSCs and the Role of MSC-EVs as Drug-Delivery Vesicles

The studies above suggest the immunomodulatory effects of MSC-EVs in various liver diseases, and in this section, we will discuss the advantages of MSC-EVs over MSCs in applications of clinical therapies and the applications of MSC-EVs as drug-delivery vesicles.

Compared to MSCs, which would be trapped in the pulmonary capillary network when administered intravenously (155, 156), leading potential pulmonary embolism (157), EVs are much smaller and can even pass-through blood-brain barriers (BBB) (158), reducing the loss of therapeutic material and the risk of treatment. Second, the cell viability of MSCs will decrease in cryogenic storage during transport. However, EVs can remain biologically active for 24 hours at 4°C (159) and for at least 7~14 days at -80°C (160, 161). Furthermore, MSC treatment requires hundreds of millions of cells, which usually takes approximately 10 weeks in cell culture, bringing a high cost of time and money (162). Comparatively, EVs can be acquired continuously and efficiently in large quantities through 3D culture systems and other methods (162). Moreover, there may be a risk of tumorigenesis after administration of MSCs, which does not occur in EVs (163). However, there is no reported clinical evidence of MSC-induced tumors due to the inefficiency of MSC transformation *in vivo*. Therefore, MSC-EVs are more feasible and appealing than MSCs as a clinical treatment in the future.

The low immunogenicity of EVs make them ideal natural vesicles compared with synthetic vesicles (164). EVs also have many advantages over other natural vesicles. First, intrinsic proteins and genetic materials in EVs indicate that EVs are capable of loading similar but exogenic biomaterials effectively. In addition, EVs are distributed widely in biological fluids such as blood, urine, and breast milk, which shows their good tolerance in the human body. Moreover, EVs can transport their cargo to target cells across the plasma membrane (165). Most importantly, EVs have the ability to target, which can be enhanced by membrane modification (166). MSCs have become an ideal source of exosomes due to their immunosuppressive effects and the ability to produce exosomes efficiently in large quantities (167). MSC-EVs are widely applied as therapeutic vehicles in liver diseases. In studies of liver fibrosis, MSC-EVs were used to carry various RNAs and proved to have antifibrotic effects, reduce ECM deposition, and improve liver function. These exogenic RNAs include miR-181-5p (168), miR-122 (169), siRNA-STAT3 and mASO-STAT3 (170). Additionally, in AIH, MSC-EVs carrying dexamethasone (DEX) could target macrophages in the liver, therefore increasing the distribution of DEX in liver tissues and leading to DEX reduction in circulation. As a result, the anti-inflammatory therapeutic effect of DEX was enhanced while its side effects were reduced in mice with ConA-induced AIH (171).

## Limitations and Improvements in Applications of MSC-EVs

Although MSC-EVs have shown great potential for immunomodulation and tissue regeneration, there are some limits in EV treatment that we should pay attention to.

Issues of heterogeneity and short half-life have hindered the application of EVs in clinical treatment. MSCs are derived from various tissue sources, resulting in different types of MSC-EVs. Although EVs from different types of MSCs exhibit similar morphology, phenotype, and function, proteomic system analysis indicates variability, which influences the therapeutic effects of MSC-EVs (172). Furthermore, recent studies have shown that the molecular composition of EVs is not cell-type dependent only but is also different in the same parental cell (173). This suggests that the molecular heterogeneity of EVs is the result of the subcellular origin of exosomes and donor cell activation status (174). However, ultracentrifugation, as the most commonly used strategy in the isolation of EVs, cannot categorize the properties of individual vesicles. To solve this problem, many improvements in EV isolation technology have emerged, such as fluorescent labeling and subsequent quantitative and qualitative analysis by high-resolution flow cytometry (175), the development of dedicated flow cytometry (176), and laser tweezers Raman spectroscopy (177). However, there is still no way to effectively eliminate this heterogeneity to meet the criteria in clinical therapy.

Another limitation is the short half-life of EVs (2 minutes to 30 minutes) (178–180) and quick clearance by the mononuclear phagocyte system (MPS) (181). Recent studies have focused on the local delivery of EVs through tissue engineering, which could be a potential strategy to enhance their feasibility. Hydrogels are polymeric networks with three-dimensional structures capable of absorbing large amounts of water or biological fluids (182). Hydrogels provide spatial and temporal control over the release of various therapeutic agents, including molecular drugs and cells (183). Due to the features of high tissue-like water content, easy implantation, and high biocompatibility, hydrogels have been increasingly used as carriers of EVs for tissue engineering. The strategy of using hydrogels as carriers for MSC-EVs has been widely used in bone (184), cartilage (185), skin (186), the nervous system (187), and the heart (188). Hydrogels showed good biocompatibility and excellent mechanical properties. Furthermore, hydrogels could release EVs continuously, enhance the stability of EVs, and improve therapeutic effects. Mardpour et al. applied tetra-PEG hydrogels to encapsulate MSC-EVs and then injected them upon intraperitoneal injection into TAA-induced chronic liver injury (CLI) mice. They reported that restricted EVs were sustained to be released over 4 weeks by gradual biodegradation and swelling of hydrogels. Conversely, free-EVs in controls were eliminated from the blood and liver within 24 hours. By prolonging bioavailability, MSC-EVs encapsulated in tetra-PEG hydrogels improved the antifibrotic, anti-apoptic, anti-inflammatory, and regenerative effects of EVs by almost 50%, compared to Free-EVs (189).

## The Applications of MSCs and MSC-EVs in Therapeutic Trials

Currently, MSCs are widely used in therapeutic clinical trials, especially in neurological (190), joint (191) and cardiovascular systems (192). In addition, several studies have focused on the therapeutic effects of MSCs in COVID-19 (193). In clinical trials focusing on liver diseases, both allogenic and autologous MSCs were used and bone marrow-derived MSCs were most commonly used, followed by umbilical cord-derived MSCs and adipose-derived mesenchymal stem cells (**Table 2**). Intravenous injection is the most commonly used method for delivering MSCs to the liver. Besides, some research chose hepatic arterial injection. Moreover, one study delivered MSCs to the liver through intrahepatic injection (196). Although these therapeutic trials used different cell types and delivery routes, they all demonstrated the safety of MSC treatments, and MSC treatment showed improvements in liver function and clinical symptoms. However, there are still some issues to be solved, such as the most effective doses *via* different routes (209).

**Table 2:** Clinical safety and efficacy of MSC therapy in liver diseases.

Clinical trials	Design	Type of cells, dose, and delivery route	participants	Follow-up	Results
Human mesenchymal stem cell transfusion is safe and improves liver function in acute-on-chronic liver failure patients (11)	An open-labeled, parallel-controlled, phase I/II trial	-Allogenic UC-MSCs -0.5 × 10^6^ UC-MSCs per kilogram -IV	43 ACLF patients -UC-MSC-treated group (n = 24) -Control (n = 19)	12 months	-Safety of UC-MSC transfusion in ACLF patients; -UC-MSC transfusions reduced the MELD score, improved liver function and alleviated liver damage for ACLF Patients
Mesenchymal stem cell therapy in decompensated liver cirrhosis: a long-term follow-up analysis of the randomized controlled clinical trial (194)	A prospective, open-labeled, randomized controlled study	-Allogenic UC-MSCs -0.5 × 10^6^ UC-MSCs per kilogram -IV	-219 patients with HBV-related decompensated liver cirrhosis -UC-MSC-treated group (n = 108) -Control (n = 111)	13~75 months	-No significant side effects or treatment-related complications were observed in the UC-MSC group -UC-MSC treatment markedly improved liver function
Bone Marrow Mesenchymal Stem Cells in Acute-on-Chronic Liver Failure Grades 2 and 3: A Phase I-II Randomized Clinical Trial (195)	A double blind, placebo-controlled, Phases I and II, randomized clinical trial	-Allogenic BM-MSCs -1.0 × 10^6^ UC-MSCs per kilogram -IV	-9 cirrhotic patients -BM-MSC treated group (n = 4) -Control (n = 5)	90 days	- BM-MSC infusion was safe, without significant side effects - BM-MSC treatment improved liver function
Transplantation with GXHPC1 for Liver Cirrhosis: Phase 1 Trial (196)	A single-center, open-labeled study	-Autologous AD-MSCs -1.0 × 10^8^ AD-MSCs/participants -Intrahepatic Injection	-6 cirrhotic patients	6 months	-Administration of AD-MSCs can be considered safe for patients with liver cirrhosis. - AD-MSC treatment improved liver function, METAVIR scores, Child–Pugh scores, MELD score and the quality of life of the cirrhotic patients
Improvement of liver function in liver cirrhosis patients after autologous mesenchymal stem cell injection: a phase I-II clinical trial (197)	A phase I-II clinical trial	-Autologous BM-MSCs -3.0~5.0 × 10^7^ BM-MSCs/participants -IV	-8 patients [hepatitis B (n = 4), hepatitis C (n=1), alcoholic (n=1), and cryptogenic (n = 2)] with end-stage liver disease having Model for End-Stage Liver Disease score ≥10	6 months	-Treatment was well tolerated by all patients - BM-MSC treatment improved liver function
Effects of allogeneic mesenchymal stem cell transplantation in the treatment of liver cirrhosis caused by autoimmune diseases (198)	A single-center, open-labeled study	-Allogenic UC-MSCs/BM-MSCs/CB-MSCs -1.0 × 10^6^ MSCs per kilogram -IV	-26 patients with liver cirrhosis caused by autoimmune Diseases - UC-MSC treated group (n = 23) -BM-MSC treated group (n = 1) -CB-MSC treated group (n = 2)	24 months	-Allogeneic MSC treatment through the peripheral vein probably was safe - Allogeneic MSC treatment improved liver function
Human umbilical cord mesenchymal stem cells improve liver function and ascites in decompensated liver cirrhosis patients (199)	A single-center, open-labeled study	-Allogenic UC-MSCs -0.5 × 10^6^ UC-MSCs per kilogram -IV	-45 patients with HBV-related decompensated liver cirrhosis -UC-MSC-treated group (n = 30) -Control (n = 15)	12 months	-UC-MSC transfusion was clinically safe - UC-MSC treatment improved liver function and reduced the volume of ascites
Allogeneic bone marrow-derived mesenchymal stromal cells for hepatitis B virus-related acute-on-chronic liver failure: A randomized controlled trial (200)	A prospective, open-label, nonblinded randomized clinical trial	-Allogenic BM-MSCs -1~10 × 10^5^ BM-MSCs per kilogram -IV	-110 patients with HBV-related ACLF -BM-MSC treated group (n = 56) -Control (n = 54)	24 weeks	-Peripheral infusion of allogeneic bone marrow–derived MSCs is safe for patients with HBV-related ACLF -BM-MSC treatment increases the 24-week survival rate by improving liver function and decreasing the incidence of severe infections
Transplantation with autologous bone marrow-derived mesenchymal stem cells for alcoholic cirrhosis: Phase 2 trial (12)	A multicenter, randomized, open-label, phase II trial	-Autologous BM-MSCs -5.0 × 10^7^ BM-MSCs/participants -hepatic arterial injection	-55 patients with alcoholic cirrhosis -18 in the control group -18 in the one-time autologous BM-MSC group -19 in the two-time autologous BM-MSC group	12 months	Autologous BM-MSC transplantation safely improved histologic fibrosis and liver function in patients with alcoholic cirrhosis
Randomized trial of autologous bone marrow mesenchymal stem cells transplantation for hepatitis B virus cirrhosis: regulation of Treg/Th17 cells (201)	A randomized, open-label, clinical trial	-Autologous BM-MSCs - 0.75 ± 0.50 × 10^6^ BM-MSCs/participants -hepatic arterial injection	-56 patients with hepatitis B virus cirrhosis -BM-MSC treated group (n = 27) -Control (n = 29)	24 weeks	-MSC transplantation further improved liver function -BM-MSC treatment increased Treg cells and decreased Th17 cells
Peripheral vein infusion of autologous mesenchymal stem cells in Egyptian HCV-positive patients with end-stage liver disease (202)	A prospective, randomized study	-Autologous BM-MSCs - 1.0 × 10^6^ BM-MSCs per kilogram -IV	-40 patients with post-HCV end-stage liver disease -BM-MSC treated group (n = 20) -Control (n = 20)	6 months	-BM-MSC treatment through the peripheral vein was safe -BM-MSC treatment improved liver functions and ascites
Phase II trial: undifferentiated versus differentiated autologous mesenchymal stem cells transplantation in Egyptian patients with HCV induced liver cirrhosis (203)	A randomized, phase II clinical trial	-Autologous BM-MSCs (Undifferentiated and differentiated respectively) - 1.0 × 10^6^ BM-MSCs per kilogram -IV	-25patients with HCV induced liver cirrhosis -undifferentiated BM-MSC treated group (n = 9) -differentiated BM-MSC treated group (n = 6) -Control (n=10)	6 months	-BM-MSC treatment through the peripheral vein was safe -BM-MSC treatment improved liver functions and ascites
Autologous bone marrow-derived cell transplantation in decompensated alcoholic liver disease: what is the impact on liver histology and gene expression patterns? (204)	A prospective, randomized study	-Autologous BM-MSCs - 0.47 ± 0.15 × 10^8^ BM-MSCs per kilogram - hepatic arterial injection	-58 patients with decompensated alcoholic liver disease -BM-MSC treated group (n = 28) -Control (n = 30)	3 months	With the negative results from the clinical trial, the impact of the BM-MSC treatment has to be interpreted as weak, and it is not able to modify the clinical course of this severe liver disease
Randomized placebo-controlled trial of mesenchymal stem cell transplantation in decompensated cirrhosis (205)	A randomized, placebo-controlled trial	-Autologous BM-MSCs - A median of 195 million (range: 120-295 million) BM-MSCs per kilogram -IV	-27 patients with decompensated liver cirrhosis -BM-MSC treated group (n = 28) -Control (n = 30)	12 months	-Autologous bone marrow MSC transplantation through peripheral vein probably has no beneficial effect in cirrhotic patients
Allogeneic bone marrow mesenchymal stem cell transplantation in patients with UDCA-resistant primary biliary cirrhosis (206)	A single-center, open-labeled study	- Allogenic BM-MSCs - A median of 195 million (range: 120-295 million) BM-MSCs per kilogram - hepatic arterial injection	-10 patients with ursodeoxycholic acid (UDCA)-resistant primary biliary cirrhosis (PBC)	12 months	- No transplantation-related side effects were observed - BM-MSC treatment improved liver functions and the quality of life -the percentage of CD8+ T cells was reduced, while that of CD4+CD25+Foxp3+ T cells was increased in peripheral lymphocytic subsets. Serum levels of IL-10 were elevated
Pilot study of umbilical cord-derived mesenchymal stem cell transfusion in patients with primary biliary cirrhosis (207)	A single-arm, open-labeled study	-Allogenic UC-MSCs -0.5 × 10^6^ UC-MSCs per kilogram -IV	-7 PBC patients with a suboptimal response to UDCA treatment	48 weeks	-UC-MSC transfusion *via* a peripheral vein is safe -UC-MSC treatment reduces the ALP and GGT levels, and improves clinical symptoms including fatigue and pruritus
Histological improvement following administration of autologous bone marrow-derived mesenchymal stem cells for alcoholic cirrhosis: a pilot study (208)	A single-center, open-labeled study	-Autologous BM-MSCs - 5 × 10^7^ BM-MSCs/participants - hepatic arterial injection	-12 patients with alcoholic cirrhosis	12 weeks	-No significant complications or side effects were observed -According to the Laennec fibrosis system, histological improvement was observed in 6 of 11 patients (54.5%).

UC-MSC, umbilical cord-derived mesenchymal stem cell; BM-MSC, bone marrow-derived mesenchymal stem cells; AD-MSC, adipose-derived mesenchymal stem cells; CB-MSC, cord blood-derived MSC; IV, Intravenous; ACLF acute-on-chronic liver failure; HBV, hepatitis B virus.

Clinical trials of MSC-EV treatments are relatively few and mostly in progress. A prospective nonrandomized open-label cohort study focused on the safety and efficacy of allogeneic bone marrow MSC-EVs as a treatment for severe COVID-19 (210). In this research, the authors reported that no adverse events were observed within 72 h of MSC-EV intravenous injection, and MSC-EV treatment improved oxygenation, downregulated the cytokine storm, and reconstituted immunity. Another study also verified the safety of nebulized clinical-grade (211). However, there is no registered clinical research on clinical trials focusing on MSC-EVs in liver diseases.

## Conclusions

MSCs play an important role in immune modulation, and recent research has suggested that the functions of MSCs are partly but significantly dependent on paracrine effects and secretory components. MSC-EVs, as the main component of MSC paracrine substances, have shown functions in immune modulation. The liver is the core metabolic and immune organ in the human body, and the loss of immune homeostasis can lead to various liver diseases. Recently, there have been many studies on MSC-EV therapy in liver diseases. However, there is little research deeply exploring the mechanism. Some research revealed the potential immunosuppressive effects of MSC-EVs in liver diseases through the modulation of macrophages and T cells, while the immune reaction and immune regulation of the liver is a comprehensive process of various innate and adaptive immune responses. Thus, it is necessary to explore whether MSC-EVs influence other immune cells, such as DCs, NK cells and B cells and how they modulate the intercellular communication between immune cells in various liver diseases.

Due to the characteristics of small size, relative stability and low immunogenicity, MSC-EVs are regarded as ideal drug-delivery vesicles and have shown therapeutic effects in preclinical research. However, there is still a long journey to clinical applications, mainly because of the issues of heterogeneity and short half-life. Currently, there is no registered clinical research on clinical trials focusing on MSC-EVs in liver diseases. We expect MSC-EVs to serve as an effective clinical therapy for liver diseases in the future.

## Author Contributions

RW and XF have organized the original manuscripts. All authors contributed to the article and approved the submitted version.

## Funding

This work was supported by the National Natural Science Foundation of China (No. 82070582 to LY) and the 1.3.5 project for disciplines of excellence, West China Hospital, Sichuan University (No. ZYGD20012 to LY).

## Conflict of Interest

The authors declare that the research was conducted in the absence of any commercial or financial relationships that could be construed as a potential conflict of interest.

## Publisher’s Note

All claims expressed in this article are solely those of the authors and do not necessarily represent those of their affiliated organizations, or those of the publisher, the editors and the reviewers. Any product that may be evaluated in this article, or claim that may be made by its manufacturer, is not guaranteed or endorsed by the publisher.

## Abbreviations

MSC, mesenchymal stem cell; EV, extracellular vesicle; NAFLD, non-alcoholic fatty liver disease; DC, dendritic cell; NK, natural killer; HLA, human leukocyte antigens; IFN-γ, interferon-γ; TNF-α, tumor necrosis factor-α; TLR, Toll-like receptor; LPS, lipopolysaccharide; ISEV, International Society for Extracellular Vesicles; Hsp, Heat-shock protein; IL, Interleukin; TGF-β, transforming growth factor β; PBMC, Peripheral blood mononuclear cell; APC, antigen-presenting cell; LSEC, hepatic sinusoidal endothelial cell; KC, Kupffer cell; APP, acute-phase protein; CRP, C-reactive protein; SAA, serum amyloid A; NKT, Natural killer T; HBV, hepatitis B virus; HCV, hepatitis C virus; Tfh, follicular helper T; Treg, regulatory T; Tc, cytotoxic T; PGE2, Prostaglandin E2; 15d-PGJ2, 15deoxy-Δ12,14-prostaglandin J2; DAMPs, damage-associated molecular patterns; HMGB1, high mobility group protein 1; NLRP3, NOD-like receptor family, pyrin domain containing 3; NAFL, nonalcoholic fatty liver disease; NASH, nonalcoholic steatohepatitis; AIH, autoimmune hepatitis; MPO, myeloperoxidase; FXR, farnesoid X receptor; ALT, alanine aminotransferase; AST, aspartate aminotransferase; MyD, myeloid differential protein; TRAF6, TNF receptor associated factor 6; AMPK, AMP-activated protein kinase; CYP2D6, cytochrome P450 2D6; ConA, Concanavalin A; HGF, hepatocyte growth factor; ALF, acute liver failure; ECM, extracellular matrix; HSC, hepatic stellate cell; α-SMA,α-smooth muscle actin; MMP, matrix metalloproteinase; IRI, ischemia–reperfusion injury; ROS, reactive oxygen species; BBB, blood-brain barrier; DEX, dexamethasone; MPS, mononuclear phagocyte system.
